# Breastfeeding in the first six months of life for babies seen by Lactation Consulting*

**DOI:** 10.1590/1518-8345.3538.3412

**Published:** 2021-04-12

**Authors:** Bruna Alibio Moraes, Juliana Karine Rodrigues Strada, Vanessa Aparecida Gasparin, Lilian Cordova do Espirito-Santo, Helga Geremias Gouveia, Annelise de Carvalho Gonçalves

**Affiliations:** 1 Universidade Federal do Rio Grande do Sul, Porto Alegre, RS, Brazil.; 2 Scholarship holder at the Coordenação de Aperfeiçoamento de Pessoal de Nível Superior (CAPES), Brazil.; 3 Universidade do Estado de Santa Catarina, Departamento de Enfermagem, Chapecó, SC, Brazil.; 4 Universidade Federal do Rio Grande do Sul, Departamento de Enfermagem Materno Infantil, Porto Alegre, RS, Brazil.

**Keywords:** Breast Feeding, Lactation, Consultants, Survival Analysis, Nursing, Nursing Care, Aleitamento Materno, Lactação, Consultores, Análise de Sobrevida, Enfermagem, Cuidados de Enfermagem, Lactancia Materna, Lactancia, Consultores, Análisis de Supervivencia, Enfermería, Atención de Enfermería

## Abstract

**Objective:**

to identify breastfeeding patterns, survival of exclusive breastfeeding and factors associated with its interruption, in the first six months of life of babies seen by Lactation Consulting.

**Method:**

a prospective cohort, with 231 mother-babies in a Baby-Friendly Hospital. An initial questionnaire was applied after 24 hours of birth, after consulting, as well as a follow-up questionnaire, applied by telephone at 15, 30, 60, 120 and 180 days, with sociodemographic and obstetric variables, maternal habits, father’s schooling, birth data and baby feeding. Survival Analysis was carried out.

**Results:**

at 180 days of age, exclusive breastfeeding was 12.7% and the probability was 19.6% in the analysis of the survival curve. The factors associated with its interruption were smoking during pregnancy (HR 1.66; CI 1.05 - 2.61), age ≥ 35 years old (HR 1.73; CI 1.03 - 2.90), difficulty in breastfeeding after hospital discharge (HR 2.09; CI 1.29 - 3.41), search for professional assistance (HR 2.45; CI 1.69 - 3.54) and use of a pacifier (HR 1.76; IC 1.21 - 2.58).

**Conclusion:**

lactation consultancy contributed to the improvement of the exclusive breastfeeding rates, although there are opportunities for advances.

## Abstract (pt)

**Objetivo:**

identificar padrões de amamentação, sobrevida do aleitamento materno exclusivo e fatores associados à sua interrupção, nos seis primeiros meses de vida de bebês atendidos por Consultoria em Lactação.

**Método:**

coorte prospectiva, com 231 mãe-bebê em Hospital Amigo da Criança. Utilizou-se questionário inicial aplicado após 24 horas do nascimento, depois da consultoria, e de seguimento, aplicado por telefone aos 15, 30, 60, 120 e 180 dias, com variáveis sociodemográficas, obstétricas, hábitos maternos, escolaridade paterna, dados do nascimento e alimentação do bebê. Procedeu-se à Análise de Sobrevivência.

**Resultados:**

aos 180 dias de vida, o aleitamento materno exclusivo foi 12,7% e probabilidade de 19,6% na análise da curva de sobrevivência. Os fatores associados à sua interrupção foram tabagismo na gestação (HR 1,66; IC 1,05 - 2,61), idade ≥ 35 anos (HR 1,73; IC 1,03 - 2,90), dificuldade na amamentação pós-alta hospitalar (HR 2,09; IC 1,29 - 3,41), busca por auxílio profissional (HR 2,45; IC 1,69 - 3,54) e uso de chupeta (HR 1,76; IC 1,21 - 2,58).

**Conclusão:**

a consultoria em lactação contribuiu para melhoria das taxas de aleitamento materno exclusivo, embora existam oportunidades de avanços.

## Abstract (es)

**Objetivo:**

identificar patrones de lactancia materna, supervivencia de la lactancia materna exclusiva y factores asociados a su interrupción, en los primeros seis meses de vida de los bebés atendidos por Consultoría de Lactancia.

**Método:**

cohorte prospectiva, con 231 madres-bebés en el Hospital Amigo del Niño. Se aplicó un cuestionario inicial a las 24 horas del nacimiento, previa consultoría, y de seguimiento, aplicado por teléfono a los 15, 30, 60, 120 y 180 días, con variables sociodemográficas, obstétricas, hábitos maternos, educación del padre, datos de nacimiento y alimentación del bebé. Se llevó a cabo un Análisis de Supervivencia.

**Resultados:**

a los 180 días de vida, la lactancia materna exclusiva fue del 12,7% y la probabilidad del 19,6% en el análisis de la curva de supervivencia. Los factores asociados a su interrupción fueron el tabaquismo en la gestación (HR 1,66; IC 1,05 - 2,61), edad ≥ 35 años (HR 1,73; IC 1,03 - 2,90), dificultad para amamantar después del alta hospitalaria (HR 2,09; IC 1,29 - 3,41), búsqueda de asistencia profesional (HR 2,45; IC 1,69 - 3,54) y uso de chupete (HR 1,76; IC 1, 21-2,58).

**Conclusión:**

la consultoría en lactancia contribuyó a la mejora de las tasas de lactancia materna exclusiva, aunque existen oportunidades de avances.

## Introduction

It is a consensus that breast milk is the most complete food for the baby, especially in the first months of life, as it is a source of nutrients in adequate amounts for a developing organism. In addition to being nutritious, breast milk protects against infections, avoids hospitalizations and reduces morbidity from diarrhea and respiratory infections, prevents episodes of otitis media and asthma, and decreases mortality from sudden childhood death syndrome. It has positive effects during adulthood, such as an increase in the intelligence quotient, education and income, as well as it protects against overweight and obesity^(^ 1 ^)^.

The benefits of breastfeeding also extend to the nursing mother. It is estimated that the expansion of breastfeeding to an almost universal level is capable to preventing 20,000 deaths *per* year of women with breast cancer, in addition to protecting the lactating woman against ovarian cancer and type 2 diabetes^(^ 1 ^)^.

The World Health Organization (WHO) adopts definitions of breastfeeding (BF) standards that are recognized worldwide^(^ 2 ^)^, which are also used in Brazil. Exclusive breastfeeding (EBF) is defined so when the child receives only breast milk or human milk from another source, without receiving other liquids or solids. When fruit juices, water or water-based drinks are introduced, BF is classified as predominant (PBF). Complementary breastfeeding (CBF) is characterized when the child receives any solid or semi-solid food for the purpose of complementing, rather than replacing, breast milk. Finally, mixed breastfeeding (MBF) occurs when other types of milk are introduced than just breast milk.

The increase in the EBF rates has a strong impact on reducing child deaths, estimating that those who are exclusively breastfed have only 12% of the risk of death compared to those who have not been breastfed^(^ 1 ^)^. Due to its benefits and the reasons for contraindicating the early introduction of other foods, the WHO and the Ministry of Health recommend BF for two years or more, being exclusively for the first six months of the child’s life^(^ 2 ^)^.

Although the benefits of breastfeeding for the mother-baby binomial and incentive policies are known, the rates remain below the recommended at the baby’s sixth month of life^(^ 3 ^-^ 4 ^)^. An important strategy to increase the number of children breastfed for the recommended period is lactation consultancy, composed of professionals qualified to provide care to the mother-baby pairs and their families in the management of difficulties with BF^(^ 5 ^)^. The lactation consultant is certified by the International Board of Lactation Consultant Examiners (IBLCE), after passing an exam offered annually in several countries^(^ 6 ^)^.

A number of studies indicate that the initiation of breastfeeding and its rates, including EBF, are positively affected by the performance of lactating consultants^(^ 7 ^-^ 8 ^)^, in addition to showing that the women seen by these professionals breastfeed for a longer period when compared to those who did not have this intervention^(^ 9 ^)^.

When considering that the first days after birth are a period in which the greatest concerns about breastfeeding occur and in which women are more susceptible to difficulties in BF^(^ 10 ^)^, and given the impact of the lactation consultant on the promotion, protection and support of breastfeeding, added to the scarcity of national studies on the performance of this professional, the present study aims to identify breastfeeding patterns, EBF survival and factors associated with its interruption, in the first six months of life for babies seen by lactation consultants.

## Method

This is a prospective cohort, carried out with mother-baby pairs seen by Lactation Consulting of the Clinical Hospital of Porto Alegre/RS (*Hospital de Clínicas de Porto Alegre*, HCPA). As a Child-Friendly Hospital (*Hospital Amigo da Criança*, HAC), the institution has since 1996 a lactation consultancy team composed, during the period of this study, by two nurses and a nutritionist, only one of the nurses with exclusive dedication to this function.

Daily, the assistance team requests, via a computerized system, support for mother-baby pairs at risk for the occurrence of breastfeeding difficulties or who are already facing problems, aiming at evaluation, support and continuous help in situations that may culminate in the interruption of the EBF before the recommended period or in early weaning. Once this request is made via the computerized system, after the appointment, the registration in the postpartum’s electronic medical record is carried out.

Pairs residing in Porto Alegre or in its metropolitan region who provided a telephone number for later contact with premature babies (gestational age defined by the Capurro method ≥ 37 weeks) and birth weight ≥ 2,500 grams, which were in joint accommodation and which had initiated breastfeeding and were seen by one of the lactation consultants. Mothers with twins, pairs with permanent or temporary contraindication for BF or who were separated after starting breastfeeding were excluded.

Participants were included from August 2016 to May 2017, with follow-up via telephone until November 2017. The sample was selected from Monday to Friday at the HCPA Obstetric Inpatient Unit (OIU), after identifying the pairs that met the inclusion criteria. The women were invited to participate in the study and, in case of acceptance and after reading it, they signed the Free and Informed Consent Form in two equal copies, one being in possession of the interviewee and the other in possession of the researcher.

For the sample calculation, a risk rate of 1.48 and a mean percentage of EBF survival of 5% at the end of six months were used, according to a study on the “maternal age below 20 years old” variable ^(^ 11 ^)^. Considering a power of 80% and a significance level of 5%, the sample size estimate was 210 mother-baby pairs. Estimating 10% losses, the sample consisted of 231 mother-baby pairs. The software used for the sample calculation was WINPEPI, version 11.43.

Two types of instruments were used to collect data. The initial, with 34 questions, and the follow-up, with 47 questions, both elaborated by the authors of the study, based on the compilation of published articles that contemplated the theme^(^ 11 ^,^ 12 ^,^ 13 ^)^ and, thus, provide a basis for achieving the objectives. These instruments were composed of closed and open questions. Data collection was carried out by two researchers who were MS students and two undergraduate Nursing students, scientific initiation scholarship holders, previously trained to approach the participants.

The instruments were applied in six stages: in person at the OIU (initial interview), which took place after the first 24 hours of the baby’s birth and after being seen by one of the lactating consultants, and via telephone at 15, 30, 60, 120 and 180 days of the baby’s life. The researchers were instrumentalized in the application of follow-up questionnaires by telephone, proceeding to read the questions previously stipulated, aiming at standardizing collection. The maximum period for applying the questionnaires was considered to be up to two days after the child completed the ages established for each follow-up. The follow-up interviews took place while the children were in BF or until contact with the participant was interrupted due to not answering the phone or due to a change of phone number during the data collection period.

They used primary data, obtained from data collection and that comprised the database of the largest research. Variables that answered to the objectives proposed in this study were used. The dependent variable was EBF interruption in days of the child’s life. The independent variables covered sociodemographic, obstetric and prenatal characteristics, maternal habits, breastfeeding history, baby feeding, difficulties in breastfeeding, reasons for not breastfeeding, offering artifacts to the baby (pacifiers, bottles, cup, spoon and syringe), support in breastfeeding, partner’s schooling and birth data.

Data was analyzed using descriptive and analytical analysis. Survival analysis was used to assess the time until EBF interruption in the first six months of the child’s life. Data on the mother-baby pairs that were still in EBF at the end of the 180-day follow-up were censored, as well as the data of the pairs that were lost during the follow-up.

To compare the characteristics of the pairs that composed the sample until the end of the study and those whose monitoring was interrupted, the Student’s *t* test was used in order to compare means; the Mann-Whitney test to compare medians; and the Chi-square test or Fisher›s exact test to compare proportions. An association was made between the variables and the time for EBF interruption through bivariate and multivariate analysis using the Cox Proportional Hazard Regression model. The median EBF time was calculated using the Kaplan-Mayer method and the curves were compared using the Log Rank test. The criterion used for the insertion of the variable in the multivariate model was that it had a p-value<0.20 in the bivariate analysis. The significance level was 5% (p≤0.05) and the analyses were performed using the SPSS program, version 21.0.

The project to which the present study is linked was approved by the Research Ethics Committee, under opinion No. 1,569,774/2016. The development of this research followed the Regulatory Guidelines and Rules for Research involving Human Beings, as established by Resolution No. 466/2012 of the National Health Council.

## Results

The study included 231 mother-baby pairs for follow-up up to 180 days of the baby’s life. Due to the impossibility of telephone contact, 18 pairs were considered loss at 15 days, 22 at 30 days, 20 at 60 days, 11 at 120 days and two at 180 days, totaling 73 (31.6%) losses up to 180 days of baby’s life, thus, 158 mother-baby pairs remaining with complete follow-up until the period stipulated for follow-up.

Table 1 shows the sociodemographic, obstetric and prenatal (PN) characteristics, maternal habits, breastfeeding history, partner’s schooling and birth data of the pairs that made up the sample until the end of the study and those whose monitoring was interrupted. These investigated variables did not display statistical significance (p≤0.05), which shows that the groups were similar, guaranteeing the quality of the analysis, even in view of the high percentage of losses. The high percentage of women who attended the minimum number of six prenatal consultations (91.6%) was evidenced, without having received information about breastfeeding during the consultations or in a group of pregnant women (62.3%). It should also be noted that the majority of the women were primiparous (67.9%), and that of those with two children or more, 59.4% said they had breastfed their last child for less than six months.

**Table 1:** Sociodemographic, obstetric and prenatal characteristics, maternal habits, breastfeeding history, partner's schooling and birth data of the 231 mother-baby pairs. Porto Alegre, RS, Brazil, 2017

Variables	Sample (n=158) n (%)	Losses (n=73) n (%)	p-value
Maternal age			0.405
< 20 years old	36 (22.8)	15 (20.5)	
20 to 35 years old	103 (65.2)	53 (72.6)	
≥ 35 years old	19 (12.0)	5 (6.8)	
Self-declared skin color			0.578
White	97 (61.4)	42 (57.5)	
Black/Brown	61 (38.6)	31 (42.5)	
Years of complete schooling of the woman			0.600
< 8 years	32 (20.3)	17 (23.3)	
≥ 8 years	126 (79.7)	56 (76.6)	
Working outside the home			0.485
Yes	68 (43.0)	35 (47.9)	
No	90 (57.0)	38 (52.1)	
Has a partner			0.360
Yes	139 (88.0)	61 (83.6)	
No	19 (12.0)	12 (16.4)	
Years of complete schooling of the partner			0.219
< 8 years	24 (15.2)	11 (15.1)	
≥ 8 years	107 (67.7)	50 (68.5)	
Did not know how to inform	8 (5.1)	0 (0.0)	
Lives with*			
Partner	129 (92.8)	56 (91.8)	0.804
Mother	36 (22.8)	21 (28.8)	0.327
Mother-in-law	19 (13.7)	3 (4.9)	0.069
Family income^†^			0.860
< 2 minimum wages	47 (29.7)	22 (30.1)	
2 to 4 minimum wages	66 (41.8)	28 (38.4)	
> 4 minimum wages	45 (28.5)	23 (31.5)	
Prenatal consultations^‡^			0.657
< 6 consultations	13 (8.4)	7 (10.3)	
≥ 6 consultations	141 (91.6)	61 (89.7)	
Information on breastfeeding in the prenatal and/or group			0.580
Yes	61 (38.6)	23 (31.5)	
No	95 (60.1)	49 (67.1)	
Did not do prenatal/group	2 (1.3)	1 (1.4)	
Smoking during pregnancy			0.166
Yes	26 (16.5)	7 (9.6)	
No	132 (83.5)	66 (90.4)	
Drug use during pregnancy			0.948
Yes	2 (1.3)	1 (1.4)	
No	156 (98.7)	72 (98.6)	
Breastfeeding time of the last child			0.092
No previous child	108 (68.4)	49 (67.1)	
< 6 months	34 (21.5)	10 (13.7)	
≥ 6 months	16 (10.1)	14 (19.2)	
Route of birth			0.778
Vaginal	94 (59.5)	42 (57.5)	
Cesarean section	64 (40.5)	31 (42.5)	
Gender of the baby			0.123
Female	63 (39.9)	37 (50.7)	
Male	95 (60.1)	36 (49.3)	
Baby classification^§^			0.280
AGA^||^	127 (80.4)	60 (82.2)	
SGA^¶^	11 (11.0)	8 (11.0)	
BGA**	20 (12.7)	5 (6.8)	

* The results can exceed 100% as there may be more than one answer;

† According to the 2017 minimum wage (R$ 954,00);

‡ Disregarding women who did not have a prenatal booklet;

§ According to Lubchenco's Curve^(^ 14 ^)^;

|| AGA = Adequate for gestational age;

¶ SGA = Small for gestational age;

** BGA = Big for gestational age

Up to the 15^th^ day after birth, all the women who participated in this stage said they had some type of support for carrying out household chores and/or baby care. In the same period, only one (0.5%) woman reported having returned to work.

Table 2 contains data regarding the interviews that took place after hospital discharge. As for the information related to hospitalization, collected at 15 days of life, it was verified that 98.1% of the women had BF complications. The use of milk formula was frequent in babies in joint accommodation (53.5%), with 42 (37.2%) women justifying its use due to the difficulty in the BF technique, 39 (34.5%) claimed the low production of milk/late support, 15 (13.3%) women reported that the reason for using milk formula was due to nipple fissure, 12 (10.6%) women attributed the use of the formula to the baby’s weight loss, and 10 (8.8%) women mentioned that the baby received a milk supplement on admission for showing hypoglycemia.

**Table 2:** Information reported by the mothers on breastfeeding patterns and related aspects, after hospital discharge and up to the baby's sixth month of life. Porto Alegre, RS, Brazil, 2017

Variables	15 days (n = 213)	30 days (n = 191)	60 days (n = 171)	120 days (n = 160)	180 days (n = 158)
	n (%)	n (%)	n (%)	n (%)	n (%)
Baby on breastfeeding					
Yes	209 (98.1)	173 (90.6)	141 (82.5)	109 (68.1)	101 (65.9)
No*	4 (1.9)	18 (9.4)	30 (17.5)	51 (31.9)	57 (36.1)
Reasons^†^					
Milk dried/Low production	3 (75.0)	7 (50.0)	4 (33.3)	-	1 (16.7)
Difficulty in the technique	1 (25.0)	5 (35.7)	2 (16.7)	1 (4.8)	6 (100)
Baby's disinterest	-	1 (7.1)	5 (41.7)	10 (47.6)	3 (50.0)
Engorgement	-	1 (7.1)	-	-	-
Baby bottle started	-	1 (7.1)	-	-	-
Anatomy of the nipple	-	2 (14.3)	2 (16.7)	1 (4.8)	-
Maternal absence	-	-	1 (8.3)	-	3 (50.0)
Allergy	-	-	-	1 (4.8)	-
Professional guidance	-	-	-	2 (9.5)	-
Breastfeeding on demand					
No	49 (23.4)	39 (22.5)	21 (14.9)	18 (16.5)	7 (6.9)
Yes	160 (76.6)	134 (77.5)	120 (85.1)	91 (83.5)	94 (93.1)
Baby receives^†^					
Tea	10 (4.7)	47 (24.6)	56 (32.7)	61 (38.1)	65 (41.1)
Water	1 (0.5)	9 (4.7)	27 (15.8)	54 (33.8)	69 (43.7)
Juice	-	-	-	14 (8.8)	36 (22.8)
Other milk	66 (31.0)	80 (41.9)	78 (45.6)	81 (50.6)	76 (48.1)
Baby receives liquids by means of^†^					
Baby bottle	62 (89.9)	92 (95.8)	97 (97)	98 (98)	81 (93.1)
Cup/Spoon/Syringe	12 (17.4)	7 (7.3)	4 (4)	3 (3)	2 (2.3)
Family support for BF^‡^	166 (79.4)	115 (60.2)	86 (50.3)	61 (38.1)	48 (30.4)
Baby using pacifier	91 (42.7)	110 (57.6)	122 (71.3)	131 (81.9)	135 (85.4)
Difficulty in BF^‡^	183 (85.9)	120 (62.8)	81 (47.4)	66 (41.25)	32 (20.3)
Smoking^§^	16 (7.5)	17 (8.9)	15 (8.8)	12 (7.5)	8 (5.1)

* Cumulative percentage over the follow-ups;

† The values may exceed 100%, as there could be more than one answer;

‡ BF = Breastfeeding;

§ 15 women already had the habit of smoking during pregnancy and only one woman reported having started after being discharged from hospital

With regard to the baby’s feeding guidance at hospital discharge, most of the mothers (80.3%) were instructed by the professionals to remain exclusively on breastfeeding, while 42 (19.7%) obtained guidance to remain on BF supplemented with milk formula. It should be noted that 91 (42.7%) babies started using a pacifier in the period between birth and the 15^th^ day of life.

**Figure 1:**
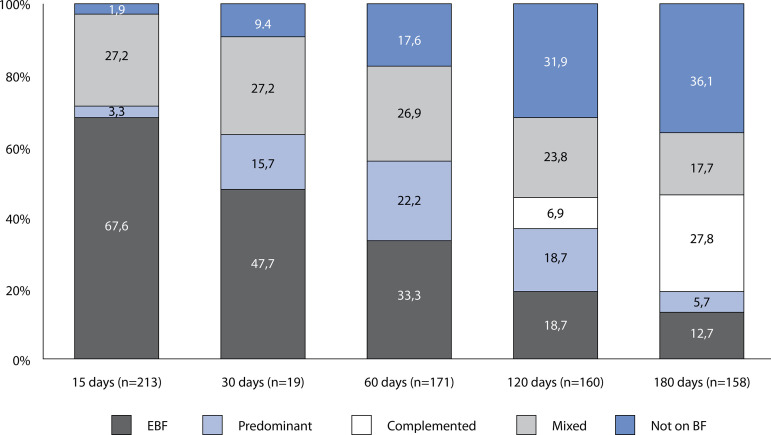
Breastfeeding patterns in the first six months of life for babies seen by a lactation consultant. Porto Alegre, RS, Brazil, 2018

It was observed that only 20 (12.7%) babies were in EBF on the 180^th^ day of life and that 57 (36.1%) had already been weaned (Figure 1). There was an increase of 21.1% in the percentage of babies on EBF at 15 days, because those babies that received dairy supplements during hospital stay, due to clinical need, and who resumed EBF at home, were not considered on EBF at the hospital stay, but on EBF at 15 days.

**Figure 2:**
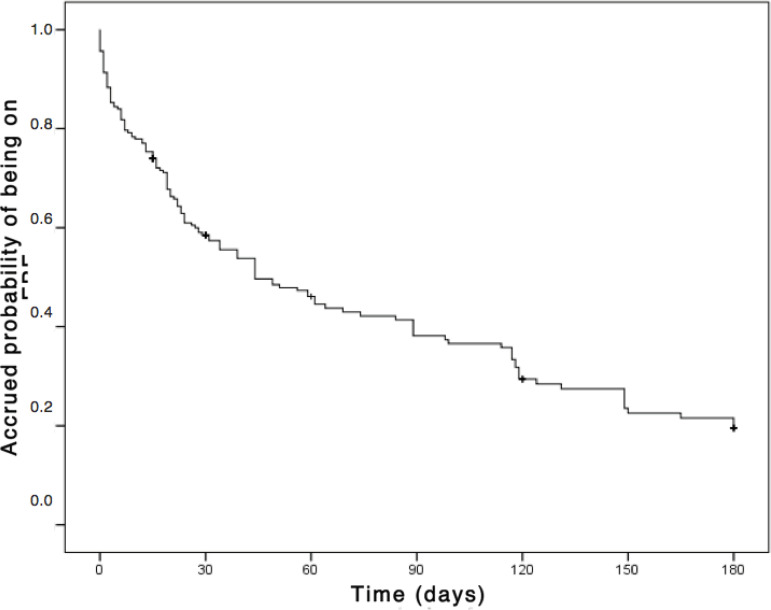
Kaplan Meier’s survival curve referring to the exclusive breastfeeding time of babies seen by a lactation consultant. Porto Alegre, RS, Brazil, 2018

Figure 2 shows the survival curve. At 15 days the probability of the baby being on EBF is 74%, with a reduction of 26% in this percentage, when compared to birth. Comparing to the immediately previous periods of follow-up, at 30 days, the probability is 58.5% with a drop of 15.5%; at 60 days, 46.1% probability with a drop of 12.4%; at 120 days, 29.4% probability with a drop of 8.8%; and at 180 days, 19.6% probability with a drop of 3.0%.

Table 3 shows the independent variables that were associated with the EBF interruption outcome up to 180 days of life in the bivariate analysis. The results show that women aged 35 years old and older, women who were smoking during pregnancy, those who had difficulty in breastfeeding after discharge and until the end of the follow-up period, and also those who sought professional assistance due to difficulties in breastfeeding after leaving the hospital are associated with a higher risk for EBF interruption before they complete 180 days of the baby’s life (p≤0.05). In addition, babies who received a pacifier after discharge also presented a higher risk of early EBF interruption.

**Table 3:** Bivariate and multivariate analysis of the factors associated with EBF interruption in infants with up to 180 days of life. Porto Alegre, RS, Brazil, 2018

Variables	Bivariate HR (95% CI)	p-value	Multivariate HR (95% CI)	p-value
Vaginal delivery	0.71 (0.51 - 0.98)	**0.039**	0.84 (0.56 - 1.24)	0.375
Baby classification*				
AGA^†^	1.00		1.00	
SGA^‡^	0.86 (0.46 - 1.59)	0.621	1.01 (0.54 - 1.89)	0.974
BGA^§^	1.60 (0.98 - 2.60)	**0.059**	1.49 (0.89 - 2.50)	0.132
White color	1.01 (0.72 - 1.41)	0.976	-	-
Maternal age				
< 20 years old	0.94 (0.62 - 1.42)	0.767	0.92 (0.57 - 1.46)	0.711
20 to 34 years old	1.00		1.00	
≥ 35 years old	181 (1.10 - 2.97)	**0.019**	1.73 (1.03 - 2.90)	**0.037**
Having a partner	0.95 (0.59 - 1.54)	0.844	-	-
Living with				
Partner	1.41 (0.66 - 3.02)	0.381	-	-
Mother	0.86 (0.58 - 1.28)	0.460	-	-
Mother-in-law	0.93 (0.54 - 1.60)	0.804	-	-
Family income				
< 2 minimum wages	1.18 (0.76 - 1.84)	0.460	-	-
2 to 4 minimum wages	1.07 (0.72 - 1.59)	0.726	-	-
> 4 minimum wages	1.00		-	-
Years of complete schooling of the woman				
< 8 years	0.97 (0.65 - 1.46)	0.898	-	-
≥ 8 years	1.00		-	-
Years of complete schooling of the partner				
< 8 years	1.13 (0.62 - 2.08)	0.689	-	-
≥ 8 years	1.00		-	-
Working outside the home	1.01 (0.73 - 1.40)	0.954	-	-
Maternity leave (months)	0.99 (0.92 - 1.06)	0.673	-	-
PN consultations^||^	0.97 (0.61 - 1.5)	0.890	-	-
< 6 consultations	1.35 (0.78 - 2.36)	0.288	-	-
≥ 6 consultations	1.00		-	-
Information on BF^¶^ in the PN^||^ or in the group of pregnant women	0.60 (0.15 - 2.49)	0.483	-	-
Smoking during pregnancy	1.68 (1.09 - 2.59)	**0.019**	1.66 (1.05 - 2.61)	**0.029**
Drug use during pregnancy	2.07 (0.51 - 8.39)	0.311	-	-
BF time^¶^ last child				
Primiparous	1.14 (0.67 - 1.93)	0.635	1.18 (0.67 - 2.06)	0.568
< 6 months	1.92 (1.05 - 3.50)	**0.033**	1.37 (0.75 - 2.49)	0.314
≥ 6 months	1.00			
Dairy complement in the hospital	1.57 (1.13 - 2.19)	**0.007**	1.34 (0.94 - 1.92)	0.107
Maternity leave at 15 days	0.95 (0.68 - 1.32)	0.756	-	-
Fixed time for BF^¶^	1.08 (0.74 - 1.59)	0.681	-	-
Pacifier use during follow-up	1.99 (1.38 - 2.87)	< 0.001*	1.76 (1.21 - 2.58)	**0.003**
Difficulty in BF^¶^ in hospitalization	0.96 (0.30 - 3.00)	0.939	-	-
Difficulty in BF^¶^ after hospital discharge	2.48 (1.54 - 3.98)	< 0.001*	2.09 (1.29 - 3.41)	**0.003**
Sought professional assistance due to breastfeeding difficulties**	2.93 (2.10 - 4.09)	< 0.001*	2.45 (1.69 - 3.54)	**< 0.001**

* According to Lubchenco's Curve^(^ 14 ^)^;

† AGA = Adequate for gestational age;

‡ SGA = Small for gestational age;

§ BGA = Big for gestational age;

|| PN = Prenatal;

¶ BF = Breastfeeding;

** Human Milk Bank, outpatient lactation consulting, primary health care

## Discussion

The survival analysis shows that the probability of the baby being in EBF decreased gradually over the follow-up period, with a greater decrease in the probability from birth to the first 15 days of the baby’s life. After 120 days there is slight stability and, after this period, the probability that the baby who reached 150 days in EBF remains this way for 180 days is greater.

It appears that the likelihood of EBF at six months (19.6%) increased nearly 3 times when compared to that obtained in a previous survey (6.6%), carried out 12 years ago, at the same institution^(^ 11 ^)^, both being cohort studies. Although this increase was significant, it still falls short of what was desired. The percentage found is also higher than that obtained in a study carried out in a Baby-Friendly Hospital in Switzerland, whose probability of EBF at 180 days was approximately 15%^(^ 3 ^)^.

This increase can reflect the public policies developed over the years in favor of BF, as well as the awareness of women and health professionals about the importance of this practice for the health of the mother-baby binomial. In addition, it can indicate greater recognition and more referrals to the lactation consultant at the institution. Furthermore, the sample of the first survey was not made up only of pairs seen by consultancy, as is the case of this one.

The breastfeeding patterns analyzed revealed a progressive reduction in EBF, as well as a gradual and continuous increase in weaning over the first six months of life of babies who were seen by lactation consultants during hospitalization. It is observed that 12.7% of the babies in the sample remained on EBF until six months of life. In comparison with the EBF rates in other cohorts, it can be seen that those found in this study are above those in other regions. In a cohort carried out in Fortaleza/CE, 65.2% of the babies were exclusively breastfed in the first month, a percentage similar to that evidenced in this study; however, the percentage drops to 3.3% in the sixth month of age^(^ 4 ^)^. In an Indian cohort, a similar trend was observed, but with a higher percentage of EBF in the first month (91.7%), reaching 11.4% of the babies breastfed exclusively until the sixth month^(^ 15 ^)^.

The aforementioned studies made no mention to the support of a lactation consulting team, which can be the justification for the EBF rates to be higher in this study. This intervention may have contributed to the increase in EBF maintenance until the babies’ sixth month of life, since receiving support and guidance on BF during hospitalization has beneficial effects on the EBF rates^(^ 7 ^-^ 8 ^)^. This shows the important role that these professionals play in maintaining exclusive breastfeeding, which has repercussions throughout the babies’ lives.

Despite these advances, when compared to the EBF rates in the sixth month of life with international cohorts, it appears that the results are below countries like Canada, with 18.5% of the babies in EBF at six months^(^ 13 ^)^, and New Zealand, with an EBF rate of 16% in the same period^(^ 16 ^)^. However, the index is above the one found in a Saudi Arabian cohort, with only 2.6% EBF at six months^(^ 12 ^)^. However, these studies were not carried out specifically with women seen by lactating consultants, which makes comparisons difficult.

In a systematic review with only randomized studies, it was verified that the interventions performed by lactating consultants have beneficial effects on the EBF rates, in addition to increasing the number of women who choose BF^(^ 8 ^)^. However, the care offered by these professionals should not be limited to the hospital environment, since the difficulties remain after returning home.

There are factors that can anticipate the introduction of other foods to the babies’ diet, which occurred more frequently between 60 and 120 days of life (14.6%). It was possible to verify that, even with the majority of women being between 20 and 35 years old at the time of the interview, those aged 35 or more had a higher risk for EBF interruption before the recommended period. These results are frequent in the literature, although controversial. Corroborating the findings, Italian researchers concluded that older mothers are less likely to breastfeed their children^(^ 17 ^)^. The opposite occurred in a study conducted in India, in which the chances of EBF increased with maternal age^(^ 18 ^)^.

As well as maternal age, smoking is also a factor frequently pointed out in the literature as interfering with EBF. A systematic review points out that non-smoking women have higher initiation and continuity of breastfeeding when compared to smokers^(^ 19 ^)^. In the present study, it was verified that 14.3% of the women were smokers during pregnancy, having a 1.66 times higher risk for EBF interruption before the baby’s six months of life, when compared to non-smokers. According to a study, most of the women who smoke during pregnancy intend to breastfeed. Despite having a high percentage of breastfeeding initiation, relapse to smoking and early weaning occurred at high rates from 12 weeks onwards, with the most cited reason for interrupting breastfeeding being the perception of insufficient milk production^(^ 20 ^)^.

However, it is noteworthy that, in the current study, 45.5% of the women smoked from pregnancy to at least the first 15 days of the baby’s life. This result was also found in a study, in which 61.7% of the women who claimed to be smoking in the postpartum period also did so during pregnancy^(^ 21 ^)^. It is known that the volume of breast milk is reduced in smokers, thus shortening the lactation period^(^ 19 ^)^. In addition, there seems to be mutual protection, since breastfeeding was considered a protective factor against increased smoking in the postpartum period. The longer the women breastfeed their babies, the less they smoke in the postpartum period, that is, investing in effective smoking cessation programs after childbirth can have a favorable impact not only on women’s health^(^ 22 ^)^.

The use of pacifiers after hospital discharge was also evidenced as a factor associated with the interruption of EBF until the babies’ sixth month of life, which is a factor frequently cited in the literature. In an analysis carried out with data from two national surveys, it was possible to conclude that the use of a pacifier was inversely associated with the EBF rates^(^ 23 ^)^. Corroborating these findings, a cohort study also reveals that using pacifiers was a risk factor for not consuming breast milk exclusively in the baby’s first month (RR = 0.90), in the 4^th^ month (RR = 1.77) and in the 6^th^ month (RR = 1.42)^(^ 24 ^)^. In addition, the use of pacifiers increased the number of unfavorable behaviors for the BF practice being considered ideal, especially regarding the body position of the mother and baby during breastfeeding, affective involvement between the mother and her child, suction efficiency and responses of the pair when initiating breastfeeding^(^ 25 ^)^.

However, there is still no consensus on the mechanism that involves the relationship between pacifier use and early BF interruption. Some authors support the hypothesis that the use of a pacifier can lead to a reduction in the number of daily feedings, causing the baby to be placed less often to breastfeed and, thus, less stimulating milk production^(^ 10 ^,^ 23 ^,^ 25 ^)^. In addition, the “nipple confusion” is also accepted by many authors as one of the explanations for the interference of this artifact in breastfeeding. A study conducted in northeastern Brazil suggests that, in the absence of other factors, such as breast trauma or difficulty in the technique, the use of artificial nipples can influence the sucking pattern, supporting the existing idea of nipple confusion^(^ 26 ^)^.

Furthermore, it is not defined in the literature whether the use of a pacifier is a marker of breastfeeding difficulties or a marker of lesser motivation to breastfeed^(^ 23 ^)^. Following this line, a recent review by the Cochrane Library on the theme concluded that the use of a pacifier, introduced before or after lactation was established, would not affect the duration of exclusive or partial breastfeeding in those mothers who were highly motivated to breastfeed their babies^(^ 27 ^)^.

Two other factors identified in this study appear to be related: women who had some difficulty in breastfeeding after hospital discharge, who had a little more than twice the risk of interrupting EBF before the baby’s six months of life, as well as those who sought help in a human milk bank, consultancy or health unit/office for support in BF due to difficulties in breastfeeding, presented an even higher risk, practically 2.5 times.

The first few days after birth can be decisive for BF success. This is a time when women are most concerned with breastfeeding and when they are more susceptible to breast problems^(^ 10 ^)^. Among such problems, breast complications stand out, which are among the main causes of food supplementation and early BF interruption^(^ 28 ^)^. This was also one of the findings of a previous cohort carried out at the same institution, in which it was possible to conclude that a poor breastfeeding technique can anticipate BF interruption in babies under six months old^(^ 11 ^)^.

These problems often lead women to seek professional help to continue breastfeeding. The active monitoring of the mothers after hospital discharge can offer an opportunity to assess and solve problems with BF, as well as direct them to community breastfeeding resources^(^ 29 ^)^. Referring the mothers to other breastfeeding support services after discharge from the maternity is essential to sustain the impacts of the HAC on long-term breastfeeding^(^ 30 ^)^. In a systematic review, the authors concluded that breastfeeding support offered to women has a positive impact on breastfeeding duration and exclusivity^(^ 31 ^)^.

However, the role of the health professional in issues related to breastfeeding seems to be a barrier to be overcome to offer quality support in post-discharge BF to the women. A study concluded that the health professionals were not sure whether the BF support offered by them was effective and complete, and trusted other colleagues to provide breastfeeding care, which resulted in problematic gaps in the guidelines on the theme^(^ 32 ^)^.

The authors suggest the development and improvement of support programs in postpartum BF that incorporate lactation consultants^(^ 8 ^)^, not necessarily in person. There are alternatives mentioned in the literature to achieve higher rates in EBF, such as counseling by certified lactation consultants, once a week, by telephone contact, starting in the third trimester of pregnancy until one week after the baby is six months old. A study concluded that the women who participated in the intervention group were more likely (97.3%) to breastfeed exclusively until six months than those in the control group (48.5%), indicating that this alternative can substantially enhance and improve practices in BF^(^ 9 ^)^.

It should be noted that the losses during the follow-up, despite not compromising the quality of the data analysis, constitute a very frequent limitation in longitudinal studies. In addition, the possibility of memory bias should also be considered, between the occurrence of the studied outcome and the interval of the connection made.

The recognition of breastfeeding patterns, the survival of EBF and the factors associated with its interruption in the first six months of life become important tools in supporting preventive strategies. With the exception of maternal age, the other factors evidenced by this study are likely to be modified. Therefore, adequate and qualified management during the pregnancy-puerperal cycle by an updated and engaged team can favor the abandonment of practices that disfavor EBF continuity.

## Conclusion

The findings showed a progressive reduction in EBF, as well as a continuous increase in weaning over the first six months of life of babies seen by lactation consultants.

The survival curve revealed that the probability of the baby being in EBF decreased gradually over the time of follow-up. However, there was a significant increase in this probability, when compared to that obtained in a study at the same institution, a little over a decade ago, which may be indicative of the important role played by lactating consultants.

The women who sought basic health units, an HMB (Human Milk Bank) and consultancy after discharge from hospital due to difficulties in breastfeeding were those who presented the greatest risk among those found for EBF interruption before the baby’s 6^th^ month of life. Therefore, the importance of working in a health network is emphasized, with support after hospital discharge, especially since it is an issue as sensitive and peculiar as BF.

It was also verified that women aged 35 years old or more had a higher risk of interrupting breastfeeding, compared to younger women, a fact that requires extra attention from the health professionals, as well as for those who were smoking during pregnancy.

Finally, the pacifier habit remains a factor linked to early EBF interruption. It is known that this is a habit strongly linked to cultural issues and it is up to the health professionals to warn against the negative contributions of its use to the baby’s health and breastfeeding.

There is a contribution of the findings of this study to advancing knowledge on the theme, since lactation consulting is an innovative strategy in health institutions, mainly because it qualifies health care for nursing mothers, babies and their families, in addition to impacting on improvements in the breastfeeding indicators.
